# Supplementary material for: Shaping Silver Nanoparticles’ Size through the Carrier Composition: Synthesis and Antimicrobial Activity
Source: Nanomaterials (Basel). 2023 May 9;13(10):1585. doi: 10.3390/nano13101585 (PMC10221138; doi:10.3390/nano13101585)
Supplement: Supplementary file 1 [file nanomaterials-13-01585-s001.zip › nanomaterials-2345459-supplementary.pdf]

Electronic supplementary information

Shaping silver nanoparticles size through the carrier composition: synthesis and antimicrobial activity

Margherita Cacaci [1,2,†], Giacomo Biagiotti [3,4,†], Gianluca Toniolo [3,4], Martin Albino [3,5], Claudio Sangregorio [5], Mirko Severi [3], Maura Di Vito [1], Damiano Squitieri [1], Luca Contiero [6], Marco Paggi [7], Marcello Marelli [8], Stefano Cicchi [3,4], Francesca Bugli [1,2,*] and Barbara Richichi [3,4,*]

[1] Dipartimento di Scienze Biotecnologiche di Base, Cliniche Intensivologiche e Perioperatorie, Università Cattolica del Sacro Cuore, 00168 Rome, Italy; margherita.cacaci@unicatt.it (M.C.); wdivit@gmail.com (M.D.V.); damiano.squitieri@unicatt.it (D.S.)

[2] Dipartimento di Scienze di Laboratorio e Infettivologiche, Fondazione Policlinico Universitario A. Gemelli IRCCS, 00168 Rome, Italy

[3] Department of Chemistry "Ugo Schiff", University of Firenze, Via della Lastruccia 13, 50019 Sesto Fiorentino, Italy; giacomo.biagiotti@unifi.it (G.B.); gianlucatoniolo.vi@gmail.com (G.T.); martin.albino@unifi.it (M.A.); mirko.severi@unifi.it (M.S.); stefano.cicchi@unifi.it (S.C.)

[4] Consorzio Interuniversitario Nazionale per la Scienza e Tecnologia dei Materiali (INSTM), 50121 Firenze, Italy

[5] ICCOM CNR, Via Madonna del Piano 10, 50019 Sesto Fiorentino, Italy; csangregorio@iccom.cnr.it

[6] Cromology Italia S.p.A., Z.I. Porcari, 55016 Lucca, Italy; luca.contiero@cromology.it

[7] IMT School for Advanced Studies Lucca, Piazza San Francesco 19, 55100 Lucca, Italy; marco.paggi@imtlucca.it

[8] CNR SCITEC-Istituto di Scienze e Tecnologie Chimiche "Giulio Natta", Via Fantoli 16/15, 20138 Milano, Italy; marcello.marelli@scitec.cnr.it

* Correspondence: francesca.bugli@unicatt.it (F.B.); barbara.richichi@unifi.it (B.R.)

† These authors contributed equally to this work.

Table of content

**Figure S1**	S3
**Figure S2**	S3
**Figure S3**	S4
**Figure S4**	S4
**Figure S5**	S5
**Table S1**	S5
**Table S2**	S6
**Table S3**	S6

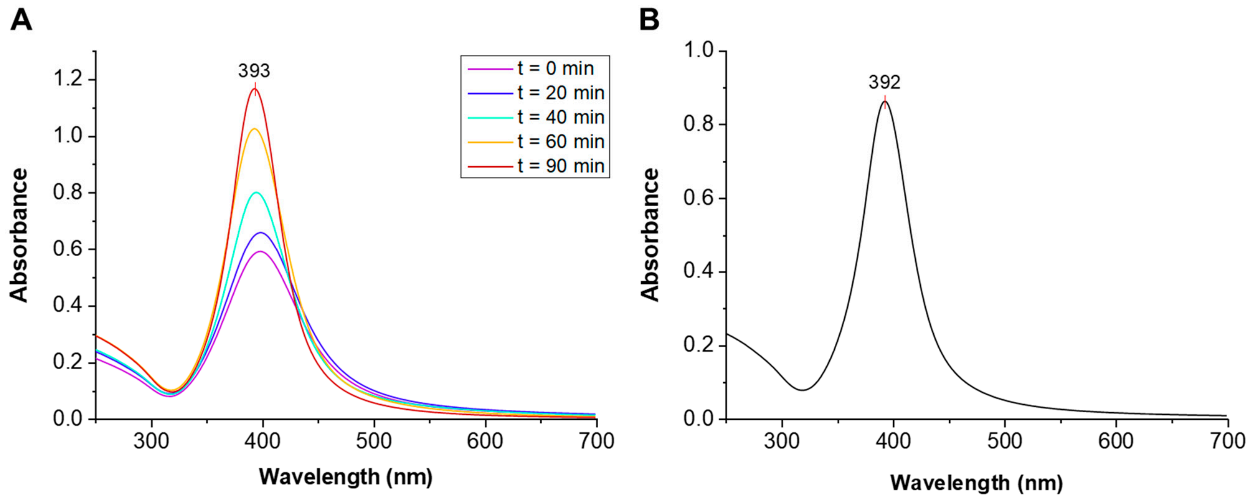

Figure S1. **A**. Overlapped UV-Vis spectra of the reaction mixture over time for the synthesis of **CNC-AgNPs**; λ_{max} = 392 nm; **B**. UV-Vis spectrum of **CNC-AgNPs** (0.13 mg/mL AgNPs in H_2O); λ_{max} = 392 nm.

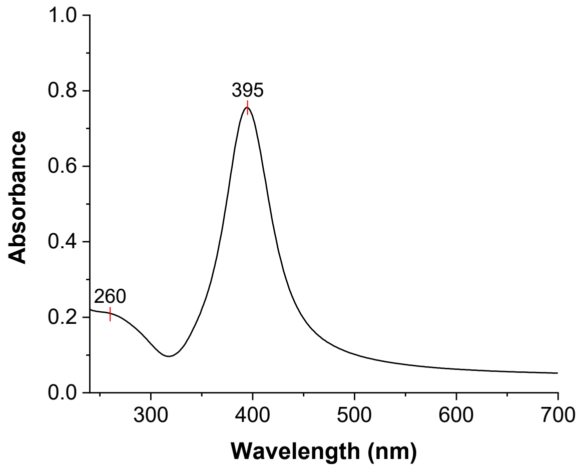

Figure S2. UV-Vis spectrum of **rGO-SA-AgNPs**. (0.1 mg/mL in H_2O); λ_{max} = 395 nm (AgNPs) λ = 260 nm (rGO-SA)

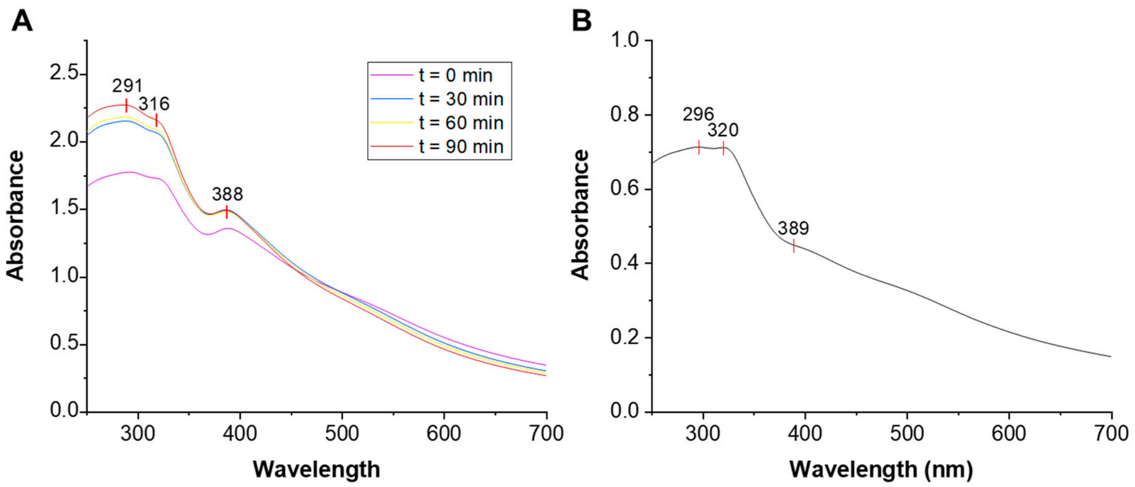

Figure S3. A) Overlapped UV-Vis spectra of reaction media for the synthesis of **TiO_2-AgNPs** (0.1 mg/mL) AgNPs λ_{max} = 388 nm; B) UV-Vis spectrum of **TiO_2-AgNPs** (0.1 mg/mL) after dialysis **(**AgNPs, λ_{max} = 389 nm), (TiO_2, λ_{max} = 316 and 291).

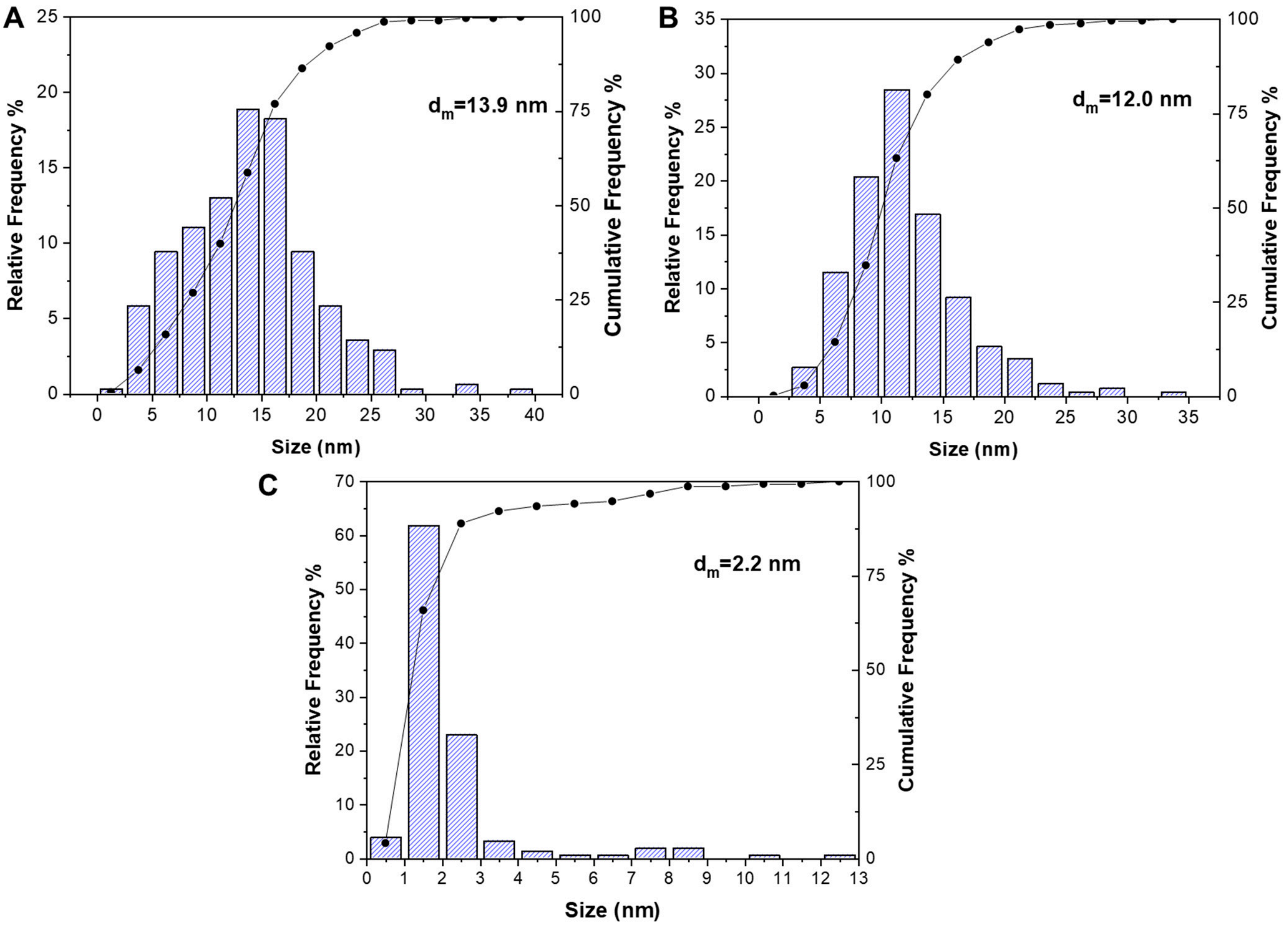

Figure S4. Nanoparticles size distribution of: A) **CNC-AgNPs**; B) **rGO-SA-AgNPs**; C**) TiO_2-AgNPs**.

Table S1. AgNPs diameter evaluated by statistical analysis of TEM images (d_{TEM}); crystal size (d_{XRD}) and lattice parameter (a) calculated by the XRD pattern fittings.

**AgNPs composites**	d_{TEM} **(nm)**[a]	d_{XRD} **(nm)**
**CNC-AgNPs**	13.93 ± 0.34	9.9 ± 0.7
**rGO-SA-AgNPs**	4.90 ± 0.27	16.2 ± 0.7
**TiO_2-AgNPs**	2.2 ± 0.2	14.2 ± 0.7

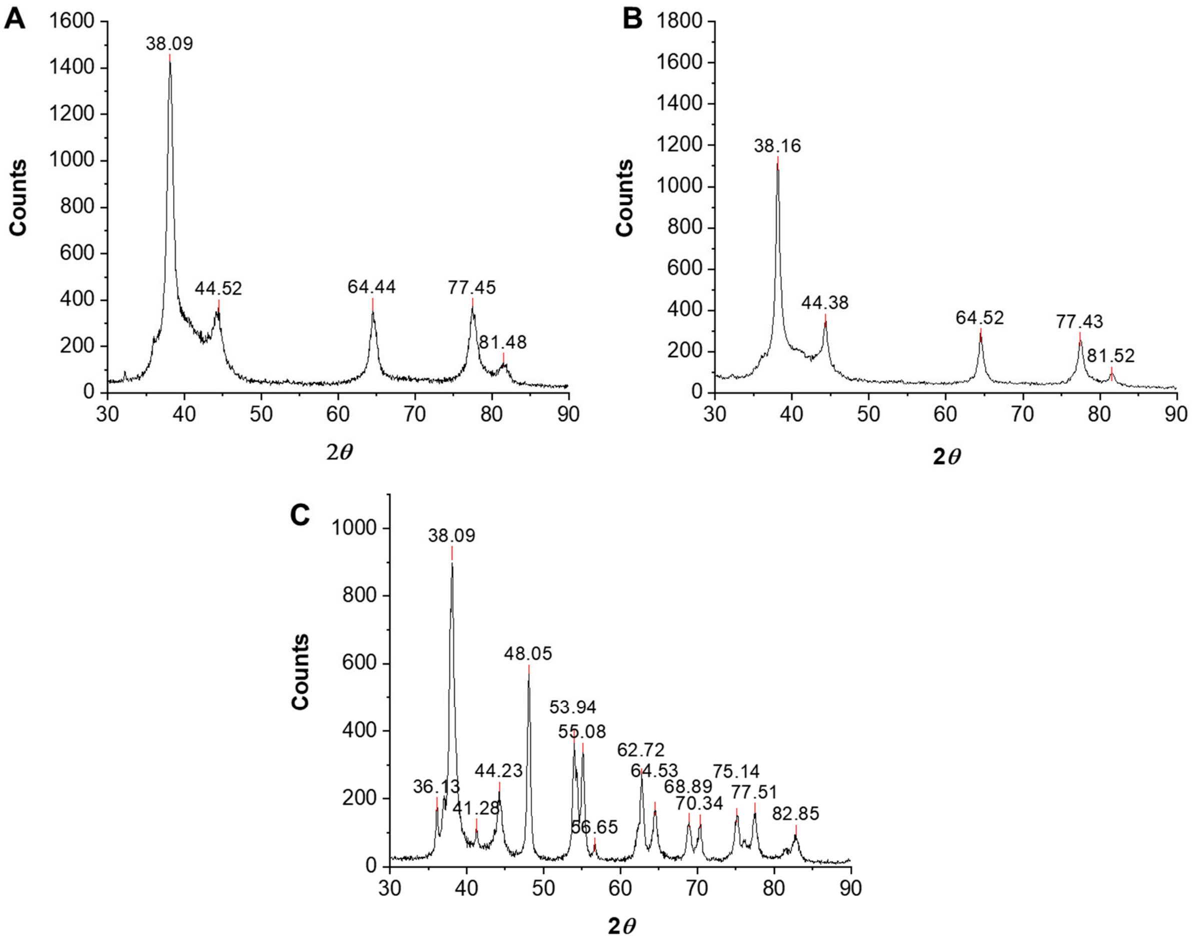

Figure S5. XRD spectra of: A) **CNC-AgNPs,** B) **rGO-SA-AgNPs**, C) **TiO_2-AgNPs**.

Table S2. Lattice parameters values (*a and c*) and crystal sizes (d_{XRD}) obtained from the Pawley refinement of the acquired XRD patterns of AgNPs (*) and TiO_2 (**) mean values and error on the last significant digit reported in brackets.

AgNPs composites	a^{*} (Å)	d_{XRD}^{*} (nm)	a^{**} (Å)	c^{**} (Å)	d_{XRD}^{**} (nm)
CNC-AgNPs	4.0849(4)	9.9(7)	-	-	-
rGO-SA-AgNPs	4.0877(3)	16.2(7)	-	-	-
TiO_2-AgNPs	4.0876(3)	14.2(7)	4.5958(9)^{e}	2.9568(7)^{e}	33.3(1)^{e}
			3.7849(4)^{f}	9.5082(8)^{f}	24.4(1)^{f}

^{e} rutile form; f anatase form.

Table S3. Silver and titanium content of the nanomaterials measured by ICP-AES and expressed as % w/w.

AgNPs composites	Ag µg/mg	Ag % w/w	Ti µg/mg	Ti % w/w
**CNC-AgNPs**	445.66	44.6	-	-
**rGO-SA-AgNPs**	1.89	18.9	-	-
**TiO_2-AgNPs**	89.4	8.9	145.74	14.6
